# Thriving from work questionnaire: Spanish translation and validation

**DOI:** 10.1186/s12889-024-18173-x

**Published:** 2024-04-27

**Authors:** Susan E. Peters, Daniel A. Gundersen, Stephanie M. Neidlinger, Jim Ritchie-Dunham, Gregory R. Wagner

**Affiliations:** 1 Center for Work, Health, and Well-being, Harvard T.H. Chan School of Public Health, Boston, MA USA; 2 Department of Social and Behavioral Sciences, Harvard T.H. Chan School of Public Health, Boston, MA USA; 3 Survey and Qualitative Methods Core, Division of Population Sciences, Dana-Farber Cancer Institute, Boston, MA USA; 4 Department of Work, Organizational, and Business Psychology, Helmut-Schmidt University, Hamburg, Germany; 5 Institute for Strategic Clarity, Belchertown, MA USA; 6 Department of Environmental Health, Harvard T.H. Chan School of Public Health, Boston, MA USA

**Keywords:** Worker well-being, Flourishing, Scale development, Measurement, Quality of life, Psychological well-being, Decent work, Healthy work design, Latin America, Psychometrics

## Abstract

**Background:**

Thriving from Work is a construct that has been highlighted as an important integrative positive worker well-being indicator that can be used in both research and practice. Recent public discourse emphasizes the important contributions that work should have on workers’ lives in positive and meaningful ways and the importance of valid and reliable instruments to measure worker well-being. The Thriving from Work Questionnaire measures how workers’ experiences of their work and conditions of work contributes in positive ways to their thriving both at and outside of work.

**Methods:**

The purpose of this study was to translate the Thriving from Work Questionnaire from English to Spanish, and then validate the translated questionnaire in a sample of 8,795 finance workers in Peru and Mexico. We used item response theory models replicating methods that were used for the original validation studies. We conducted a differential item functioning analysis to evaluate any differences in the performance of models between Peru and Mexico. We evaluated criterion validity with organizational leadership, flourishing, vitality, community well-being, and worker’s home location socio-economic position.

**Results:**

The current study demonstrates that the Spanish (Peru/Mexico) questionnaire was found to be a reliable and valid measure of workers’ thriving from work. One item was dropped from the long-form version of the original U.S. questionnaire. Both the long and short form versions of the questionnaire had similar psychometric properties. Empirical reliability was high. Criterion validity was established as hypothesized relationships between constructs was supported. There were no differences in the performance of the model between countries suggesting utility across Latin American countries.

**Conclusions:**

The current study demonstrated that the Spanish (Peru and Mexico) version of the questionnaire is both a reliable and valid measure of worker well-being in Latin America. Specific recommendations are made for the adaptation of the questionnaire and directions of future research.

**Supplementary Information:**

The online version contains supplementary material available at 10.1186/s12889-024-18173-x.

## Background

The changing nature of work, workforces, and working conditions all have significant implications for worker well-being. Throughout history, scholars have frequently noted that work can benefit well-being. Employment is an important social determinant of health, and the quality of employment and the work performed have the ability to shape people’s relationships, livelihoods, and their overall satisfaction with life. Work gives a sense of meaning and purpose, and provides the economic means to prosper.

There is a growing body of evidence that exposure to specific working conditions and workplace hazards influences well-being [1–3]. While the vast majority of research focuses on how work can be detrimental to one’s health and well-being, more attention is now being drawn towards the positive role that work can play in contributing to one’s overall quality of life. The recent COVID-19 pandemic highlighted the importance that workers were placing on their own well-being. Workers are moving to jobs where organizations are prioritizing worker well-being as a key organizational value. Workers and organizations are looking for strategies and ways to ensure that workers are thriving; workers with higher levels of well-being and happiness are less likely to turnover, are more productive and collaborative, and are healthier and therefore incurring less healthcare expenditure for chronic health conditions. This research exemplifies the importance of work as a determinant of population health and well-being.

The measurement of whether workers are thriving from their work (or not) is therefore critical as an indicator of worker well-being. Organizational data are integral for employers to better understand the needs of their workforce, and to ensure that work is designed to support worker well-being. Thus, there is a need for well-developed and valid measures of worker well-being. One such reliable and valid measure is the National Institute for Occupational Safety and Health (NIOSH) Well-being Questionnaire (WellBQ) which provides a diagnostic tool of 68 items for organizations to identify well-being needs of workers [4]. However, there are limited instruments that have been developed that assess the positive contribution that work can have on one’s ability to thrive in their lives. To address this gap, the Harvard T.H. Chan School of Public Health Center for Work, Health, and Well-being defined and conceptualized the concept of Thriving from Work, and later developed a long- (30 item) and short- (8 item) questionnaire.

The framing of the concept of “Thriving from Work” responded to repeated calls for an expanded view of occupational safety and health by measuring positive work-related well-being across several dimensions [5]. In a previous study, we conducted a series of formative studies that resulted in several products. First, they developed a definition for ‘Thriving from Work’ as “the state of positive mental, physical, and social functioning in which workers’ experiences of their work and working conditions enable them to thrive in their overall lives, contributing to their ability to achieve their full potential in their work, home, and community” ( [6]p.05). Next, we conceptualized worker well-being into six specific dimensions across 30 important attributes that have been found through formative research to be integral for positive worker well-being. These dimensions include (i) job design for supporting thriving from work, (ii) social well-being from work, (iii) work-life integration, (iv) health and physical and mental well-being at work, (v) basic needs for thriving from work, and (vi) psychological and emotional well-being from work [6 ]. From this framework, we then developed a reliable and valid Thriving from Work Questionnaire in two diverse samples of U.S. workers. This questionnaire was designed to both measure the construct of ‘thriving from work’ as well as provide a mechanism for organizations (or other entities) to identify priority areas that would benefit from interventions focused on improving worker well-being. Thus, the questionnaire enables companies to (a) identify priority areas for improving worker well-being, (b) identify groups of workers (e.g., work teams) that are thriving (or have low levels of worker well-being), and (c) measure change in levels of thriving among and between groups of workers.

Once a reliable and validated instrument was developed, there is a subsequent need to translate and validate the instrument into different languages and in different geographic regions. This is to ensure that the same constructs are measured using the same instrument in different work contexts across a range of global research and practice settings. The purpose of the present study was to translate the English version of this questionnaire to Spanish, and then validate the Spanish questionnaire in two geographical regions, Peru and Mexico, herein called the *Thriving from Work Questionnaire– Spanish P-M*.

## Methods

### Sample

8,795 workers from a large Latin American finance company based in Mexico and Peru completed a survey as part of baseline data collection for a large organizational change initiative in June-July 2021. This sample comprised 31% of the entire workforce from the finance company.

### Translation of the questionnaire

To translate the Thriving from Work Questionnaire from English to Spanish, we performed a well-accepted translation and back-translation approach using a professional translation company with two different bilingual translators as described by Beaton et al. [8] Both translated versions of the questionnaire were then reviewed by the research team, and any discrepancies were resolved through discussion with the translation team. The questionnaire was then reviewed by a bi-lingual member of our research team (JRD) and representatives from our finance company collaborators based in Mexico and Peru to ensure that the content validity of the items was retained.

### Variables and measures

All office-based employees were invited by the company leadership team through an email invitation to voluntarily complete an electronic questionnaire. We assessed the correlation of the Thriving from Work measure with other conceptually similar instruments to establish concurrent criterion validity. That is, does Thriving from Work correlate with other constructs of interest in a manner that is theoretically and conceptually expected?

### Thriving from work questionnaire

The Thriving from Work Questionnaire by Peters et al. (2021) contains six domains: (1) psychological and emotional well-being from work, (2) social well-being from work, (3) work–life integration, (4) basic needs for thriving from work, (5) job design and experience of work, and (6) safety and physical and mental well-being from work. Thirty items representing 30 attributes of Thriving from Work are measured on the long-form across the six domains (3–6 items per domain). The short-form questionnaire contains eight items representing all six domains. Respondents rated all items on a Likert scale: Always, Almost Always, Usually, Sometimes, Rarely, or Never. In two U.S. samples representing a diverse group of workers, the U.S. English version of the questionnaire was found to have excellent internal consistency and test-retest and empiral reliability, as well as content and criterion validity for both the long- and short- forms [9]. 

In this paper, we examine criterion validity using two scoring methods– both of which have been found to be appropriate and may be used depending on the research question and study design– (i) a summed or unit-weighted score across all items in the long- and short-forms, respectively; and (ii) maximum expected posteriori scores. For both methods, a higher score indicates higher thriving from work.

### Validation measures

A variety of measures were used to evaluate concurrent criterion validity. We evaluated the correlation between thriving from work and the validation measures hypothesizing the direction and strength of the relationship based on how conceptually similar or distinct the measure is in relation to thriving from work. Thus, we evaluated thriving from work with measures that were conceptually similar and hypothesized that these would have higher correlations. We also considered measures that we considered more conceptually distinct and hypothesized that these would have a lower correlation with thriving from work.

#### Organizational leadership

Organizational leadership was measured using the leadership subscale items from the Institute for Strategic Clarity’s Organizational Agreements Health Checklist, which assesses a worker’s experience of work conditions, from extractive to flourishing, in how they support the worker’s relationship to self, to another, to the group, to the group’s creative process, and to the group’s creative source [10]. This was the only external construct that we used that was tied to the workplace. Example items include: “Our leadership recognizes the gifts of all its employees, and invites each of us to express them in fulfilling our greatest potential”, “Leadership inspires us to value our differences”, and “Our leadership helps us to take into account all the stakeholders in what we do.” Reliability was ${\alpha }$= 0.96. We hypothesized that the Thriving from Work measure would be positively correlated with the Organizational Agreements Health Checklist and would have a higher correlation than the other non-work focused constructs.

#### Flourishing

The Secure Flourishing index was used to measure human flourishing, consisting of 12 items that were developed by VanderWeele [11]. It measures six domains of human flourishing: (1) happiness and life satisfaction, (2) mental and physical health, (3) meaning and purpose, (4) character and virtue, (5) close social relationships, and (6) financial and material stability. Each domain contains two items, and the sum score across all 12 items represents an overall human flourishing measure, with a higher score representing higher flourishing. Items are similar to the Thriving from Work items, except they are focused on well-being in one’s life rather than as a result of their work. Sample items are “Overall, how satisfied are you with life as a whole these days?”, “I understand my purpose in life”, and “I am content with my friendships and relationships.” Each item has an 11-point Likert scale ranging from 0 as the most negative rating to 10 as the most positive rating possible. We hypothesized that the Thriving from Work Questionairre measure would be moderately correlated with flourishing in a positive direction and that flourishing would be more strongly associated with thriving from work than community well-being. Reliability was ${\alpha }$= 0.79.

#### Meaning and purpose

Meaning and purpose was assessed using the instrument, The Comprehensive Measure of Meaning [12, 13]. The respondents rated meaning and purpose by answering 21 items from 7 domains: (1) global coherence, (2) individual coherence, (3) subjective significance, (4) objective significance, (5) direction and mission, (6) direction and purpose, and (7) direction and goals. Sample items are “I have a framework that allows me to understand or make sense of human life”, “Living is deeply fulfilling”, “I make a significant contribution to society”, and “My current aims match with my future aspirations”. Reliability was ${\alpha }$= 0.95. As meaning and purpose is an important dimension of work-related well-being– as it relates to deriving a sense of meaning and purpose from one’s work–we expected there to be moderate levels of correlation between thriving from work and meaning and purpose.

#### Vitality

Vitality was measured using the Subjective Vitality scale [14, 15]. Using seven items, responses are captured on a 5-point Likert scale response from Strongly Disagree to Strongly Agree. A higher score indicates higher vitality. Reliability of the scale was ${\alpha }$= 0.59. We expected there to be a moderate correlation between thriving from work and vitality.

#### Community Well-being

Community well-being was measured using the short-form Subjective Community Well-being measure [13]. This is a five-item instrument that captures each worker’s perspective of the extent to which they perceive the well-being of their community across five dimensions of community well-being first defined by VanderWeele et al.: good relationships (trust); proficient community leadership, satisfying community, strong community mission (purpose); and healthy community practices (achievement of community’s goals). These items were summed to create an overall score of community well-being. Reliability of the scale was ${\alpha }$= 0.93. As a thriving community is expected to be made up of thriving people who reside in that community, we expected there to be a positive correlation between a workers’ perception of their community well-being and thriving from work, but a lower correlation than we would expect for constructs that are more directly related to the workplace.

#### Workers’ home location socio-economic position

To explore whether employees’ local circumstances influence thriving from work, we conducted additional analyses with the Mexican sample using state-level publicly available data from the Mexican Instituto Nacional de Estadística y Geografía (INEGI) dataset. This included eight socio-economic variables matched to each worker’s geographical location within Mexico: percentage of victims of crime per household, the average degree of schooling of the population 15 years old and over, the percentage of the population aged 12 years and more who are economically active (employed), percentage of population receiving the minimum wage, average literacy level for the region, and the quality of housing available. Thus, each employee had variables that described their own living environment. We assumed that living in an area with high crime rates, low quality of housing, low education, and low income might hinder people from thriving from work, but at a lower level than the other validation constructs. We expected there to be a low level of correlation between their home environment and their thriving from work.

### Statistical analysis to establish psychometric properties

To assess the psychometric properties of the *Thriving from Work Questionnaire– Spanish P-M*, we first characterized the item-level responses to identify items that lacked variability or had high item-nonresponse. Second, we fit item response theory (IRT) models with the items comprising the long- and short-forms English version. Specifically, for the long-form, a bifactor-graded response model was fit with all items loading on general thriving and each item loading on the respective specific domains as identified in the English sample. We present marginal discrimination parameters for the long-form calculated by logistic approximation [16]. For the short-form, we fit a unidimensional graded response IRT model. We judged model fit using limited information chi-square statistics (M2 or C2, depending on the model), root mean squared error of approximation (RMSEA), standardized root mean square residual (SRMSR), and the comparative fit index (CFI). These measures evaluate the fit of the measurement model relative to saturated models (M2, C2; lower values indicating better model fit), absolute fit (RMSEA; lower values indicating better fit), the deviation of the model implied correlation matrix from the observed correlation matrix (SRMSR; lower values indicating better fit), and improvement in fit compared to a “null” model (CFI; higher value indicating better fit). As these are different measures, we judged overall model fit by interpreting each measure in context of the others rather than relying on strict cut-off values for each. Although the latter is a common approach, simulation studies have shown such cut-off values have substantial limitations [17–19]. The short form was modelled twice– first with “psychological safety” and then with “physical safety”– as these two items are highly correlated but one may be more relevant for some job contexts than others. We conducted a similar process when we completed the original U.S. English validation study in which we recommended that the two safety items ("physical safety" and "psychological safety" could be used interchangeably in the short-form version [9]. We present the empirical reliability as a measure of scale reliability for general thriving from work as well as for each specific domain of the long-form.

Lastly, we conducted a differential item functioning (DIF) analysis according to the method of Crane et al., to establish whether the questionnaire’s items perform similarly/differently across the two countries [20]. Specifically, we estimated modal a-posteriori factor scores for the general and specific Thriving from Work domains. We then fit a series of ordinal logistic regression models, where each item was (1) regressed on thriving from work scores, (2) regressed on thriving from work scores and a country indicator variable, and (3) regressed on thriving from work scores, a country indicator variable, and an interaction term for country and general thriving from work. We regarded non-uniform DIF if the interaction term for model 3 was significant at a Bonferroni adjusted α = 0.05. We regarded uniform DIF if the coefficient for general thriving from work was >|10%| different from model 2 relative to model 1. We did not test for DIF for the specific factors (domains) as these are, to date, not considered to all have adequate reliability for individual analyses. This process was repeated for the short-form, less the specific factor domain scores as they are not present in the short form’s unidimensional model. All analyses were implemented using the mirt (Multidimensional Item Response Theory) package in R [21].

## Results

### Description of the sample

There was a total of 8,795 employees working across multiple service offices in Latin America who completed the survey: 8,254 were in the Mexican sample, and 541 in the Peruvian sample (Table 1). The survey was completed as part of a large organizational change project and thus all data collected were included in this study. The sample was gender balanced, with 49.9% identifying as male, 49.6% identifying as female, and a small percentage identifying as gender diverse. Participants were on average 34.28 years old (SD = 6.81) in the general sample with the Mexican sample being, on average, about 4 years older than the Peruvian sample (Mexico: Mean 34.52 years old (SD = 6.79); Peru: Mean 30.64 years (SD = 5.99)). The mean tenure for the total sample was 4.89 years (SD = 3.51), ranging from less than a year to 37 years.

**Table 1:** Demographic Characteristics of Employees

Baseline characteristic	Mexican sample		Peruvian Sample		Full sample	
	*n*	%	*n*	%	*n*	%
Gender						
Female	4148	50.3	238	44.0	4386	49.9
Male	4063	49.2	300	55.5	4363	49.6
Other	43	0.5	3	0.6	46	0.5
Highest educational level						
Elementary school	3	0.0	0	0.0	3	0.0
Middle school	210	2.5	13	2.4	223	2.5
High school/some college	3144	38.1	0	0.0	3144	35.7
Associate degree	998	12.1	230	42.5	1228	14.0
Professional degree	3774	45.7	269	49.7	4043	46.0
Master’s degree	123	1.5	29	5.4	152	1.7
Doctorate	2	0.0	0	0.0	2	0.0

### Validation of the long-form

Table 2 displays the distributions for each item of the long-form. No items had ceiling or floor effects and most tended to skew towards the higher responses (i.e., Usually to Always). The exception to this pattern is “I feel excessive levels of stress from my work” (Stress), which had a near uniform distribution across the response categories. This item, "Stress", was thus dropped from the long-form for the *Thriving from Work Questionnaire– Spanish P-M* version. Additional File 1 contains the correlations of items in the Thriving from Work Questionnaire.

**Table 2:** Item distributions

Item	Never	Rarely	Sometimes	Usually	Almost always	Always
My work adds meaning to my life (Meaning)	1.1	1.2	5.5	13.3	23.1	55.7
My job allows me to achieve my full potential (Potential)	0.6	1.3	7.1	12.1	30.1	48.7
I love my job (Enthusiasm)	0.6	1.1	5.3	11.1	24.2	57.7
The kind of work I do makes me happy (Happiness)	0.4	0.8	4.1	9.0	21.4	64.4
I am satisfied with my job (Satisfaction)	0.3	0.6	3.5	8.8	22.9	63.9
My work adds to my overall life satisfaction (Life Satisfaction)	0.4	0.6	4.1	10.0	25.3	59.7
I feel supported by the people I work with (Support)	1.1	2.8	9.8	12.6	27.6	46.0
I feel valued by the people I work with (Valued)	1.7	4.4	11.1	13.5	27.7	41.7
I am treated fairly at work (Fairness)	3.7	7.1	14.6	13.6	23.0	38.1
I am treated with respect at work (Respect)	4.6	6.6	12.6	13.2	23.7	39.4
At work, I feel like I belong (Belonging)	1.5	3.4	9.0	14.6	23.6	47.9
I receive recognition at work for my accomplishments (Recognition)	0.5	1.3	4.3	10.4	18.8	64.6
I can voice concerns or make suggestions at work without getting into trouble (Voice)	0.6	1.4	5.5	12.4	22.4	57.6
I can easily manage my job as well as attend to the needs of my family (Work-Family)	1.4	3.5	9.6	15.2	26.9	43.4
I feel safe getting to and from work (Commute)	1.4	4.4	11.3	15.9	27.4	39.6
I can achieve a healthy balance between my work and my life outside of work (Work-Life)	1.0	1.9	9.9	16.1	27.0	44.0
I am paid fairly for the job I do (Pay)	1.8	2.4	9.2	15.1	24.7	46.7
I am satisfied with the amount of paid leave I can take to care for myself or family members (Benefits)	2.4	3.6	8.1	12.7	21.4	51.9
I feel my job is secure (Job Security)	1.0	1.9	7.1	12.3	23.0	54.6
I have good promotion opportunities (Progression)	5.3	9.1	11.5	16.4	21.5	36.3
I am happy with how much input I have in decisions that affect my work (Autonomy)	1.3	2.0	6.8	17.4	29.5	43.0
I have adequate control over the pace of my work (Work Intensity)	0.7	1.8	6.4	16.3	32.3	42.6
I am happy with how much control I have over my work schedule (Schedule)	0.6	1.9	7.2	15.8	30.9	43.6
I can easily manage the demands of my job (Demands)	0.3	0.8	5.3	15.8	34.4	43.4
I have access to the resources I need to do my job well (Resources)	0.4	1.6	5.0	13.8	27.8	51.4
I feel physically safe at work (Physical Safety)	0.8	1.7	7.5	14.6	27.4	48.0
I feel psychologically safe at work (Psychological Safety)	1.0	1.9	7.5	13.9	26.6	49.1
I feel excessive levels of stress from my work (Stress)	18.2	18.7	17.3	12.3	12.5	21.0
I worry that I will get hurt at work (Injury)	5.7	14.7	28.3	17.1	17.2	17.1
After I leave work, I have enough energy to do the things I want or need to do (Energy)	5.0	11.4	23.8	19.8	20.0	19.9

Table 3 presents estimated marginal discrimination parameters for general thriving as well as each specific domain. The median estimated discrimination parameter was 2.34, with a range of 0.47 to 3.63 in absolute value. Only one estimated discrimination parameter was less than 1 in absolute value (“I worry that I will get hurt at work” (Injury): -0.47), while the vast majority were well above 1. The estimated discrimination parameters were smaller for the specific domains than general Thriving from Work. The marginal discrimination parameters for Psychological and Emotional Well-being ranged from 0.67 to 1.12; Social Well-being from Work from 0.56 to 0.95; Work-life Integration from 0.32 to 1.00; Basic Needs from 0.36 to 0.55; Experience of Work & Job Design from 0.18 to 0.91; and, for Physical and Mental Well-being and Safety from − 0.24 to 0.69. Model fit were M2_(df = 234)_ = 4901.4, RMSEA = 0.05 (95% CI lower-bound = 0.05), SRMSR = 0.06, and CFI = 0.94. Empirical reliability was 0.93 for general Thriving from Work and ranged from 0.29 for Basic Needs for Thriving to 0.59 for Psychological and Emotional Well-being from Work, among the specific domains.

**Table 3:** Marginal discrimination parameters, long-form Thriving from Work Questionnaire Spanish P-M

Item	General Thriving from Work	Psychological Emotional Well-being from Work	Social Well-being from Work	Work-Life Integration	Basic Needs for Thriving	Experience of Work & Job Design	Physical & Mental Well-being and Safety
My work adds meaning to my life (Meaning)	1.76	0.75					
My job allows me to achieve my full potential (Potential)	2.15	0.67					
I love my job (Enthusiasm)	2.12	0.99					
The kind of work I do makes me happy (Happiness)	2.16	1.12					
I am satisfied with my job (Satisfaction)	2.31	0.95					
My work adds to my overall life satisfaction (Life Satisfaction)	2.44	0.88					
I feel supported by the people I work with (Support)	2.32		0.82				
I feel valued by the people I work with (Valued)	2.4		0.95				
I am treated fairly at work (Fairness)	1.9		0.71				
I am treated with respect at work (Respect)	2.26		0.69				
At work, I feel like I belong (Belonging)	2.52		0.56				
I receive recognition at work for my accomplishments (Recognition)	2.38						
I can voice concerns or make suggestions at work without getting into trouble (Voice)	3.63						
I can easily manage my job as well as attend to the needs of my family (Work-Family)	2.52						
I feel safe getting to and from work (Commute)	2.34			0.75			
I can achieve a healthy balance between my work and my life outside of work (Work-Life)	2.19			1.00			
I am paid fairly for the job I do (Pay)	1.88			0.32			
I am satisfied with the amount of paid leave I can take to care for myself or family members (Benefits)	1.94				0.55		
I feel my job is secure (Job Security)	2.62				0.50		
I have good promotion opportunities (Progression)	1.98				0.36		
I am happy with how much input I have in decisions that affect my work (Autonomy)	2.62				0.36	0.20	
I have adequate control over the pace of my work (Work Intensity)	2.55					0.82	
I am happy with how much control I have over my work schedule (Schedule)	2.48					0.91	
I can easily manage the demands of my job (Demands)	2.47					0.56	
I have access to the resources I need to do my job well (Resources)	2.62					0.18	
I feel physically safe at work (Physical Safety)	2.37						0.65
I feel psychologically safe at work (Psychological Safety)	2.93						0.69
I worry that I will get hurt at work (Injury)	-0.47						-0.24
After I leave work, I have enough energy to do the things I want or need to do (Energy)	1.44						0.16
Empirical reliability	0.93	0.59	0.58	0.54	0.29	0.52	0.39

Model fit: M2_(df=234)_ = 4901.4; RMSEA = 0.05 (95%CI-LB = 0.05); SRMSR = 0.06; CFI = 0.94

### Validation of the Short-form

For the short-form with “psychological safety” to represent the safety attribute, the estimated discrimination parameters ranged from 1.79 (“I am treated fairly at work” [Fairness]) to 2.56 (“I am happy with how much input I have in decisions that affect my work” [Autonomy]) (See Table 4). Model fit was C2_(df=20)_ = 729.9, RMSEA = 0.06 (95%CI lower-bound = 0.06), SRMSR = 0.05, and CFI = 0.99. The empirical reliability for the short form was 0.87. The short-form with “physical safety” measuring the safety attribute resulted in discrimination parameters that were very similar, empirical reliability was also 0.87 and model fit was C2_(df=20)_ = 1228.8, RMSEA = 0.08 (95%CI lower-bound = 0.08), SRMSR = 0.05, and CFI = 0.97. Thus, the discrimination parameters and model fit indices were comparable regardless of which item was used in the short-from.

**Table 4:** Short-form discrimination and intercepts

Item	Discrimination Parameter	Category Intercepts				
		1	2	3	4	5
I love my job (Enthusiasm)	2.31	7.43	6.13	4.23	2.60	0.59
I am treated fairly at work (Fairness)	1.79	4.50	3.14	1.76	0.83	-0.68
I can achieve a healthy balance between my work and life outside of work (Work-Life)	2.18	6.55	5.25	3.18	1.61	-0.37
I am paid fairly for the job I do (Pay)	1.84	5.42	4.41	2.82	1.47	-0.16
I am happy with how much input I have in decisions that affect my work (Autonomy)	2.56	6.91	5.75	4.04	1.98	-0.46
I can easily manage the demands of my job (Demands)	2.53	8.44	6.94	4.61	2.35	-0.47
I can voice my concerns or make suggestions at work without getting into trouble (Voice)	3.21	9.02	7.37	5.23	3.12	0.78
I feel psychologically safe at work (Psychological Safety)*	2.91	7.85	6.40	4.29	2.41	-0.01

Empirical reliability = 0.87.

Model fit: C2_(df=20)_ = 729.9; RMSEA = 0.06 (95%CI-LB = 0.06); SRMSR = 0.05; CFI = 0.98

* This item can be swapped with the item, “I feel physically safe at work” as both items performed similarly in the model. That is, in the short-form questionnaire, the Physical Safety and Psychological Safety items performed similarly in the models. This means that either one of these items could be used interchangeably in the questionnaire at the discretion of the researcher. Please refer to the Methods and Results section for more information

### Differential item functioning by country

No items were identified as having uniform DIF for the long- or short-forms. For the long-form, one item, “physical safety”, had evidence of non-uniform DIF with a p-value below the threshold (*P* = 0.0004) but it had practically similar associations with general thriving from work (b = 3.65 for Mexico vs. 3.10 for Peru). For uniform DIF, all changes in the coefficient for general thriving from work were less than 1%. For the short-form, the smallest p-value for non-uniform DIF was for the “valued” item (*P* = 0.072) DIF all items had less than 1% change in the coefficient for general thriving from work.

### Relationship with external variables

To establish criterion validity, in the long-form we found that thriving from work was positively correlated with organizational leadership (*r* = 0.60), meaning and purpose (*r* = 0.46), vitality (*r* = 0.44), flourishing (*r* = 0.41), and community well-being (*r* = 0.31) in an order of magnitude as hypothesized (Table 5). As expected, as the construct moved conceptually and theoretically further away from being influenced by one’s work and in relation to one’s own well-being the association became smaller. Organizational agreements health and general flourishing–being the most conceptually similar–had the highest associations with thriving from work, whereas community well-being, an evaluation of the well-being of one’s community, had the lowest association. Likewise, the short-form had similar positive correlations with the external conceptually similar constructs, and these were almost identical to the long-form correlations. All correlations were significant on the *p* < 0.001 level.

**Table 5:** Criterion validity: Pearson correlations

	Expected a posteriori scores		Sum scores	
	TfW long	TfW short	TfW long	TfW short
TfW short	0.97	-	-	0.97
Company Leadership	0.60	0.57	0.63	0.60
Meaning and Purpose	0.46	0.47	0.49	0.48
Vitality	0.44	0.45	0.48	0.46
Flourishing	0.41	0.41	0.42	0.40
Community Well-being	0.31	0.29	0.29	0.28

We found that the Thriving from Work Questionnaire also had very weak correlations between the socio-economic position of a worker’s home location across the 8 factors examined (between ß = − 0.06 and ß = 0.09, ns). This suggests that thriving from work captures the strong relationship that work has with well being, and is minimally affected by external contextual factors outside of work.

## Discussion

The Thriving from Work Questionnaire measures the unique positive contribution that work has for one’s well-being both at work and life outside of work. Overall, this study found that the *Thriving from Work Questionnaire– Spanish P-M* version is a robust, reliable, and valid instrument that can serve as a measure of work-related well-being with some specific recommendations for its use in Spanish and deployment for surveys in research and practice.

### Reliability and model fit

We reported marginal discrimination parameters ranging from 0.66 to 2.92 in our original validation paper. The current study had marginal discrimination parameters on par with the original validation paper [9] and thus were considered good. This current Spanish validation study also found the instrument to have good construct and criterion validity, and good reliability. Indeed, the measurement model that was identified in our original validation study [9] was also found to have good fit for both the Spanish version long- and short-forms in this present study. Specifically, although the M2 or C2 statistics were significant, the RMSEA indicated an acceptable level of misfit, the SRMSR found that the deviation of the model implied and observed correlation matrix differed by a negligible degree, and the CFI indicated a substantial improvement when compared to a model with zero covariances among the variables. Overall, when considering all the information that these model-fit statistics provided, they support that the model has good fit. Likewise, the reliability, as indicated by the empirical reliability statistic, was high, and, although we observed higher item-level scores on most items, the conditional standard error of measurement was good across the range of the Thriving from Work scores. In summary, the reliability coefficient, marginal discrimination parameters, and the criterion related validity were similar to what we found in the original validation studies conducted using the English version in the U.S [9] and are acceptable.

There was one item that did not perform as expected and we recommend further study to better understand its performance in the instrument (see Recommendations for Further Study and Use of the Spanish (Peru and Mexico) Questionnaire). The item–“I feel excessive levels of stress from my work” ("stress")–did not perform as expected compared to the original English language validation. Indeed, its distribution was near uniform across the response categories and its correlation with other items were generally much lower than the average correlation among the remaining variables. For those considering use of the questionnaire in Peru and Mexico, this suggests that this item may potentially be removed from the long-form without affecting its reliability or validity. Otherwise, the short and long versions of the questionnaire performed as expected.

Lastly, we also found that the questionnaire’s properties were similar when comparing Peruvian and Mexican data highlighting that this version of the questionnaire may have utility in other Latin American countries that have similar contextual characteristics as Peru or Mexico. Only one item showed some evidence of differential item functioning between the two countries, but the direction of the association with general thriving from work was the same and the magnitude similar to what we would expect. One could either apply differential scoring for this item or, if judged to be negligible for a user’s application, scoring it the same way but effectively treating it as a non-DIF item (that is, scoring it in the same way as the other items as recommended by Peters et al. [9]).

### Criterion validity

The *Thriving from Work Questionnaire - Spanish P-M* version of the instrument correlated with external constructs with similar but conceptually different characteristics as expected. The magnitude and direction of these relationships were hypothesized based on how conceptually similar or distinct these constructs were. We hypothesized that higher correlations would be observed with constructs that were more closely related to thriving from work, such as organizational leadership. Conversely, lower correlations would be observed with those constructs more conceptually distinct, such as community well-being. Our hypotheses were supported by our findings and, thus, support the criterion validity for the *Thriving from Work Questionnaire - Spanish P-M*.

We expected that the quality of organizational leadership, measured using the Organizational Health Agreements Checklist, is likely an important antecedent for thriving from work and would have the highest correlation: when the quality of organizational leadership is low than we would also expect that this would have a direct negative impact on worker well-being. Further, when leaders prioritize worker well-being, workers are more likely to thrive from their work by driving accountability for worker well-being and shaping working conditions and job demands to support worker well-being.

We hypothesized that community well-being would have the lowest association with workers’ thriving from work as it was conceptually the most different from our conceptualization of thriving from work. To compare, thriving from work is a positive wholistic worker well-being construct that evaluates how work contributes to the positive mental, physical, and social functioning of individuals. Whereas community wellbeing–an individual’s perception of their community’s well-being– is more conceptually distinct as it does not consider one’s own well-being nor is it focused on one’s work. Thus, the relationship between thriving from work and community well-being would be low.

Using data linkage, we were also able to assess whether thriving from work is associated with external contextual factors not linked to one’s own employment; we assessed workers’ home locations’ socio-economic position using state-level public data in Mexico which we linked to each worker. We found that there was minimal association with one’s home socio-economic position (such as crime rate, quality of housing, average literacy level for the region, and employment rates) on one’s own thriving from work. This provides evidence that the Thriving from Work Questionnaire measures work-related thriving and does not appear to be associated with or influenced by home socio-economic context. This has implications for researchers and practitioners using the instrument to measure the unique contribution that work has on a worker’s well-being.

### Recommendations for further study and use of the Spanish (Peru and Mexico) Questionnaire

The long and short versions of the questionnaire performed as expected, except for the performance of one of the original items within the physical and mental health and safety domain. During the analysis, one item– “stress”– did not perform as it did in the U.S. calibration and validation samples. The item “I feel excessive levels of stress from my work” ("stress") did not perform as expected (compared to the original English language validation). We suggest that the impact of workplace stress on thriving from work may be more pronounced in U.S. contexts due to differences in how working conditions manifest (such as higher workloads and blurring of work-life boundaries) and lack of regulation around psychosocial stressors, thus reducing the level of information this item adds in other cultural contexts. However, this might be due to a number of reasons, including inadequate or poor translation of the item, too high literacy level needed to interpret the translated item, or the item may be interpreted differently than the original U.S. English interpretation. Although we conducted a limited number of cognitive tests in Peru and Mexico, this may not have been sufficient to represent the vast range of education and possible literacy levels within the validation sample. At this stage, we would recommend further study of this item, and additional cognitive testing especially if being used in a different Spanish dialect. Further testing we conducted also suggests that the item could be removed from the questionnaire with limited impact on its psychometric properties.

Context matters when considering whether an instrument is valid for the specific use that is planned. Geographical region, cultural context, and translation differences across dialects of a given language may result in modifications needing to be made to a questionnaire. This Spanish validation was conducted in Mexico and Peru which have subtly different linguistic characteristics. This increases our confidence in the use of the instrument in other Latin American countries. We also currently have a validation effort underway in Chile which will further enhance our knowledge of the utility of the Thriving from Work Questionnaire in other Latin American countries. However, we do recommend a best practice approach of cognitive testing of the Spanish translation in other contexts, depending on the Spanish dialect spoken and any other contextual factors that may influence how items are interpreted.

Additionally, users of the questionnaire may want to consider whether they use the *physical safety* or *psychological safety* item when using the short-form version. This item was found to perform similarly–with respect to reliability, model fit, and validity–using either of the safety items. This means that users of the questionnaire may select which of the safety items they prefer to use in the short-form (that is, to select either the *physical safety* or *psychological safety* item), depending on which item might be more appropriate for the occupational setting. For example, psychological safety may be more relevant to office based jobs, and physical safety might be more relevant to manual jobs.

The sample used in this study was a large worker sample (*n* = 8,795) from one organization based in the finance sector. As workers are all from the same sector, there is a possibility that they may not be representative of other sectors. However, the original development and validation samples were across a diverse sample of workers. Thus, we feel that this validation, although in one sector, provides preliminary evidence for the reliability and validity of the instrument. However, future studies in different settings will enhance our confidence in the utility of the instrument and its properties. Additionally, the workers lived in geographically different regions across two countries including workers from urban and non-urban areas from more than 300 offices; all of which had different workplace characteristics such as culture, office size, leadership, and variations in the implementation of different workplace policies and practices.

## Conclusions

The Thriving from Work Questionnaire measures how workers’ experiences of their work and conditions of work contribute to their thriving both at and outside of work. The current study demonstrated that the Spanish (Peru and Mexico) version of the questionnaire is both a reliable and valid measure of worker well-being. Specific recommendations are made to the adaptation of the questionnaire and directions of future research.

### Electronic supplementary material

Below is the link to the electronic supplementary material.

Additional file 1. Thriving from Work Questionnaire - Spanish P-M Version

Additional File 2. Thriving from Work Item Correlations

## Data Availability

The data that support the findings of this study are not publicly available but reasonable requests should be addressed to the corresponding author.
