# Supplementary material for: Thriving from work questionnaire: Spanish translation and validation
Source: BMC Public Health. 2024 Apr 27;24:1187. doi: 10.1186/s12889-024-18173-x (PMC11055305; doi:10.1186/s12889-024-18173-x)
Supplement: Supplementary file 1 — Additional file 1. Thriving from Work Questionnaire - Spanish P-M Version [file 12889_2024_18173_MOESM1_ESM.docx]

**Additional File 2. Thriving from Work Item Correlations**

# APPENDIX A – ITEM INTERCORRELATIONS

| **#** | **1** | **2** | **3** | **4** | **5** | **6** | **7** | **8** | **9** | **10** | **11** | **12** | **13** | **14** | **15** | **16** | **17** | **18** | **19** | **20** | **21** | **22** | **23** | **24** | **25** | **26** | **27** | **28** | **29** |
| --- | --- | --- | --- | --- | --- | --- | --- | --- | --- | --- | --- | --- | --- | --- | --- | --- | --- | --- | --- | --- | --- | --- | --- | --- | --- | --- | --- | --- | --- |
| **2** | 0.75 |  |  |  |  |  |  |  |  |  |  |  |  |  |  |  |  |  |  |  |  |  |  |  |  |  |  |  |  |
| **3** | 0.73 | 0.78 |  |  |  |  |  |  |  |  |  |  |  |  |  |  |  |  |  |  |  |  |  |  |  |  |  |  |  |
| **4** | 0.72 | 0.75 | 0.87 |  |  |  |  |  |  |  |  |  |  |  |  |  |  |  |  |  |  |  |  |  |  |  |  |  |  |
| **5** | 0.70 | 0.75 | 0.81 | 0.88 |  |  |  |  |  |  |  |  |  |  |  |  |  |  |  |  |  |  |  |  |  |  |  |  |  |
| **6** | 0.72 | 0.77 | 0.82 | 0.86 | 0.88 |  |  |  |  |  |  |  |  |  |  |  |  |  |  |  |  |  |  |  |  |  |  |  |  |
| **7** | 0.54 | 0.63 | 0.61 | 0.62 | 0.65 | 0.66 |  |  |  |  |  |  |  |  |  |  |  |  |  |  |  |  |  |  |  |  |  |  |  |
| **8** | 0.54 | 0.62 | 0.60 | 0.60 | 0.63 | 0.63 | 0.86 |  |  |  |  |  |  |  |  |  |  |  |  |  |  |  |  |  |  |  |  |  |  |
| **9** | 0.47 | 0.56 | 0.51 | 0.51 | 0.54 | 0.55 | 0.68 | 0.74 |  |  |  |  |  |  |  |  |  |  |  |  |  |  |  |  |  |  |  |  |  |
| **10** | 0.50 | 0.58 | 0.53 | 0.53 | 0.55 | 0.57 | 0.73 | 0.77 | 0.75 |  |  |  |  |  |  |  |  |  |  |  |  |  |  |  |  |  |  |  |  |
| **11** | 0.50 | 0.56 | 0.55 | 0.54 | 0.57 | 0.57 | 0.71 | 0.76 | 0.71 | 0.81 |  |  |  |  |  |  |  |  |  |  |  |  |  |  |  |  |  |  |  |
| **12** | 0.49 | 0.52 | 0.54 | 0.54 | 0.55 | 0.55 | 0.65 | 0.68 | 0.59 | 0.70 | 0.81 |  |  |  |  |  |  |  |  |  |  |  |  |  |  |  |  |  |  |
| **13** | 0.63 | 0.67 | 0.68 | 0.71 | 0.71 | 0.71 | 0.73 | 0.77 | 0.67 | 0.74 | 0.79 | 0.78 |  |  |  |  |  |  |  |  |  |  |  |  |  |  |  |  |  |
| **14** | 0.58 | 0.63 | 0.65 | 0.66 | 0.67 | 0.69 | 0.62 | 0.64 | 0.56 | 0.61 | 0.60 | 0.57 | 0.70 |  |  |  |  |  |  |  |  |  |  |  |  |  |  |  |  |
| **15** | 0.55 | 0.61 | 0.63 | 0.63 | 0.64 | 0.66 | 0.60 | 0.61 | 0.55 | 0.59 | 0.57 | 0.52 | 0.65 | 0.84 |  |  |  |  |  |  |  |  |  |  |  |  |  |  |  |
| **16** | 0.51 | 0.56 | 0.58 | 0.58 | 0.60 | 0.61 | 0.55 | 0.56 | 0.52 | 0.57 | 0.56 | 0.54 | 0.63 | 0.66 | 0.70 |  |  |  |  |  |  |  |  |  |  |  |  |  |  |
| **17** | 0.51 | 0.59 | 0.57 | 0.59 | 0.61 | 0.63 | 0.54 | 0.53 | 0.53 | 0.51 | 0.53 | 0.49 | 0.59 | 0.56 | 0.57 | 0.59 |  |  |  |  |  |  |  |  |  |  |  |  |  |
| **18** | 0.47 | 0.52 | 0.53 | 0.54 | 0.55 | 0.56 | 0.53 | 0.53 | 0.50 | 0.53 | 0.55 | 0.54 | 0.58 | 0.59 | 0.62 | 0.58 | 0.61 |  |  |  |  |  |  |  |  |  |  |  |  |
| **19** | 0.54 | 0.60 | 0.61 | 0.61 | 0.63 | 0.64 | 0.62 | 0.63 | 0.57 | 0.62 | 0.64 | 0.61 | 0.70 | 0.63 | 0.62 | 0.61 | 0.61 | 0.65 |  |  |  |  |  |  |  |  |  |  |  |
| **20** | 0.47 | 0.58 | 0.53 | 0.53 | 0.55 | 0.57 | 0.59 | 0.62 | 0.63 | 0.64 | 0.62 | 0.55 | 0.63 | 0.56 | 0.55 | 0.54 | 0.61 | 0.55 | 0.66 |  |  |  |  |  |  |  |  |  |  |
| **21** | 0.53 | 0.62 | 0.60 | 0.61 | 0.63 | 0.64 | 0.64 | 0.65 | 0.60 | 0.64 | 0.63 | 0.60 | 0.69 | 0.61 | 0.61 | 0.59 | 0.57 | 0.57 | 0.65 | 0.66 |  |  |  |  |  |  |  |  |  |
| **22** | 0.57 | 0.63 | 0.64 | 0.65 | 0.65 | 0.67 | 0.61 | 0.61 | 0.55 | 0.59 | 0.58 | 0.56 | 0.67 | 0.72 | 0.71 | 0.63 | 0.57 | 0.59 | 0.65 | 0.58 | 0.70 |  |  |  |  |  |  |  |  |
| **23** | 0.56 | 0.63 | 0.64 | 0.65 | 0.66 | 0.67 | 0.60 | 0.60 | 0.54 | 0.57 | 0.57 | 0.55 | 0.66 | 0.71 | 0.70 | 0.62 | 0.58 | 0.59 | 0.65 | 0.57 | 0.68 | 0.86 |  |  |  |  |  |  |  |
| **24** | 0.56 | 0.61 | 0.63 | 0.65 | 0.65 | 0.66 | 0.58 | 0.57 | 0.52 | 0.56 | 0.56 | 0.56 | 0.66 | 0.66 | 0.66 | 0.62 | 0.53 | 0.57 | 0.64 | 0.54 | 0.65 | 0.76 | 0.78 |  |  |  |  |  |  |
| **25** | 0.53 | 0.60 | 0.60 | 0.60 | 0.61 | 0.62 | 0.62 | 0.60 | 0.57 | 0.62 | 0.63 | 0.61 | 0.68 | 0.61 | 0.62 | 0.61 | 0.61 | 0.60 | 0.66 | 0.60 | 0.65 | 0.68 | 0.69 | 0.71 |  |  |  |  |  |
| **26** | 0.51 | 0.56 | 0.58 | 0.58 | 0.60 | 0.60 | 0.58 | 0.57 | 0.49 | 0.57 | 0.58 | 0.57 | 0.64 | 0.62 | 0.63 | 0.71 | 0.55 | 0.59 | 0.65 | 0.56 | 0.61 | 0.65 | 0.65 | 0.67 | 0.70 |  |  |  |  |
| **27** | 0.57 | 0.63 | 0.65 | 0.64 | 0.66 | 0.67 | 0.63 | 0.65 | 0.56 | 0.63 | 0.65 | 0.64 | 0.72 | 0.69 | 0.67 | 0.67 | 0.58 | 0.61 | 0.71 | 0.60 | 0.65 | 0.70 | 0.70 | 0.71 | 0.70 | 0.79 |  |  |  |
| **28** | 0.42 | 0.47 | 0.48 | 0.49 | 0.49 | 0.51 | 0.47 | 0.48 | 0.43 | 0.47 | 0.44 | 0.38 | 0.49 | 0.60 | 0.61 | 0.52 | 0.47 | 0.51 | 0.50 | 0.47 | 0.47 | 0.56 | 0.57 | 0.53 | 0.49 | 0.52 | 0.57 |  |  |
| **29** | 0.25 | 0.22 | 0.27 | 0.27 | 0.24 | 0.24 | 0.19 | 0.20 | 0.14 | 0.19 | 0.22 | 0.23 | 0.25 | 0.29 | 0.26 | 0.21 | 0.18 | 0.25 | 0.25 | 0.15 | 0.14 | 0.23 | 0.24 | 0.23 | 0.22 | 0.25 | 0.31 | 0.19 |  |
| **30** | -0.03 | -0.11 | -0.06 | -0.07 | -0.09 | -0.10 | -0.11 | -0.11 | -0.14 | -0.09 | -0.06 | -0.01 | -0.07 | -0.13 | -0.14 | 0.01 | -0.12 | -0.05 | -0.06 | -0.14 | -0.11 | -0.13 | -0.13 | -0.10 | -0.05 | 0.06 | -0.03 | -0.18 | 0.44 |
| Note: *N* = x. Polychoric correlations displayed since individual items are to be considered ordinal variables | | | | | | | | | | | | | | | | | | | | | | | | | | | | | |

| 1 | My work adds meaning to my life |
| --- | --- |
| 2 | My job allows me to achieve my full potential |
| 3 | The kind of work I do makes me happy |
| 4 | I love my job |
| 5 | I am satisfied with my job |
| 6 | My work adds to my overall life satisfaction |
| 7 | I feel supported by the people I work with |
| 8 | I feel valued by the people I work with |
| 9 | I receive recognition at work for my accomplishments |
| 10 | I can voice concerns or make suggestions at work without getting into trouble |
| 11 | I am treated fairly at work |
| 12 | I am treated with respect at work |
| 13 | At work, I feel like I belong |
| 14 | I can achieve a healthy balance between my work and my life outside of work |
| 15 | I can easily manage my job as well as attend to my needs and the needs of my family |
| 16 | I feel safe getting to and from work |
| 17 | I am paid fairly for the job I do |
| 18 | I am satisfied with the amount of paid leave I can take to care for myself or family members |
| 19 | I feel my job is secure |
| 20 | I have good opportunities for promotion |
| 21 | I am happy with how much input I have in decisions that affect my work |
| 22 | I have adequate control over the pace of my work |
| 23 | I am happy with how much control I have over my work schedule |
| 24 | I can easily manage the demands of my job |
| 25 | I have access to the resources I need to do my job well |
| 26 | I feel physically safe at work |
| 27 | I feel psychologically safe at work |
| 28 | I worry that I will get hurt at work [R] |
| 29 | I feel excessive levels of stress from my work [R] |
| 30 | After I leave work, I have enough energy to do the things I want or need to do |

[R] Reverse Coded
