# Supplementary material for: Thriving from work questionnaire: Spanish translation and validation
Source: BMC Public Health. 2024 Apr 27;24:1187. doi: 10.1186/s12889-024-18173-x (PMC11055305; doi:10.1186/s12889-024-18173-x)
Supplement: Supplementary file 2 — Additional File 2. Thriving from Work Item Correlations [file 12889_2024_18173_MOESM2_ESM.docx]

**Thriving from Work Questionnaire - Spanish P-M Version**

**This additional file contains the Spanish version of the questionnaire that was translated for the study from the English version.**

**Suggested Introductory Text:**

The following items relate to how you perceive the work you do day-to-day.

*Indicate how often, if at all, you have generally felt that way about your work over the last month.*

*Select one response for each item.*

**Response categories:** Never, Rarely, Sometimes, Usually, Almost Always, Always

Los siguientes elementos se relacionan con la forma en la que usted percibe el trabajo que realiza a diario.

*Indique la frecuencia con la que se ha sentido de determinada manera en general en relación con su trabajo durante el último mes. Elija una respuesta para cada elemento.*

**CATEGORÍAS DE RESPUESTA:** Siempre, Casi siempre, Normalmente, A veces, Rara vez, Nunca, No se aplica

*Indicates items in Short-form.
**For the short-form, either the physical safety or psychological safety item can be used depending on the characteristics of the workers.

| **DIMENSIÓN** | **ITEM (English)** | **ELEMENTO (Español)** |
| --- | --- | --- |
| *Psychological and Emotional Well-being from Work* | 1. My work adds meaning to my life. | 1. Mi trabajo le agrega sentido a mi vida. |
|  | 1. My job allows me to achieve my full potential. | 1. Mi trabajo me permite alcanzar todo mi potencial. |
|  | 1. The kind of work I do makes me happy. | 1. El tipo de trabajo que hago me hace feliz. |
|  | 1. I love my job.* | 1. Me encanta mi trabajo. |
|  | 1. I am satisfied with my job. | 1. Estoy satisfecho con mi trabajo. |
|  | 1. My work adds to my overall life satisfaction. | 1. Mi trabajo contribuye positivamente con mi satisfacción general con mi vida. |
| *Social Well-being from Work* | 1. I feel supported by the people I work with. | 1. Me siento apoyado por la gente con la que trabajo. |
|  | 1. I feel valued by the people I work with. | 1. Me siento valorado/a por las personas con las que trabajo. |
|  | 1. I am treated fairly at work.* | 1. Me tratan de manera justa en el trabajo. |
|  | 1. I am treated with respect at work. | 1. Me tratan con respeto en el trabajo. |
|  | 1. At work, I feel like I belong. | 1. En el trabajo, siento que pertenezco. |
|  | 1. I can achieve a healthy balance between my work and my life outside of work.* | 1. Puedo lograr un equilibrio saludable entre mi trabajo y mi vida fuera del trabajo. |
|  | 1. I can easily manage my job as well as attend to my needs and the needs of my family. | 1. Puedo realizar fácilmente mi trabajo y atender mis necesidades y las de mi familia. |
| *Work-Life Integration* | 1. I feel safe getting to and from work. | 1. Me siento seguro yendo y volviendo del trabajo. |
|  | 1. I am paid fairly for the job I do.* | 1. Recibo un pago razonable por el trabajo que hago. |
|  | 1. I am satisfied with the amount of paid leave I can take to care for myself or family members. | 1. Estoy satisfecho con la cantidad de días de licencia pagada que puedo tomar para ocuparme de mí mismo o de mi familia. |
| *Basic Needs from Thriving from Work* | 1. I feel my job is secure. | 1. Creo que mi trabajo es seguro. |
|  | 1. I have good opportunities for promotion. | 1. Tengo buenas oportunidades de ascenso en el trabajo. |
|  | 1. I am happy with how much input I have in decisions that affect my work.* | 1. Me siento satisfecho/a con el nivel de aportes que contribuyo a las decisiones que afectan mi trabajo. |
|  | 1. I have adequate control over the pace of my work. | 1. Tengo un control adecuado sobre mi ritmo de trabajo. |
| *Job design and experience of work* | 1. I am happy with how much control I have over my work schedule. | 1. Estoy feliz con el control que tengo sobre mi cronograma de trabajo. |
|  | 1. I can easily manage the demands of my job. | 1. Puedo manejar fácilmente las exigencias de mi trabajo. |
|  | 1. I have access to the resources I need to do my job well. | 1. Tengo acceso a los recursos que necesito para hacer bien mi trabajo. |
|  | 1. I feel physically safe at work. | 1. Me siento físicamente seguro en el trabajo. |
|  | 1. I feel psychologically safe at work.* | 1. Me siento psicológicamente seguro en el trabajo. |
| *Health & Physical and Mental Well-being from work* | 1. I worry that I will get hurt at work. | 1. Me preocupa lastimarme en el trabajo. |
|  | 1. After I leave work, I have enough energy to do the things I want or need to do. | 1. Cuando salgo del trabajo, tengo suficiente energía para hacer las cosas que quiero o necesito hacer. |
|  | 1. I receive recognition at work for my accomplishments. | 1. Recibo reconocimiento en el trabajo por mis logros. |
|  | 1. I can voice concerns or make suggestions at work without getting into trouble. | 1. Puedo expresar mis inquietudes o hacer sugerencias en el trabajo sin meterme en problemas. |

For any further information, or if you have any questions, please contact: Dr. Susan Peters, Center for Work, Health, and Well-being, [sepeters@hsph.harvard](mailto:sepeters@hsph.harvard)
